# Strain diversity of *Borrelia burgdorferi *sensu lato in Serbia

**DOI:** 10.1186/1756-3305-7-S1-O25

**Published:** 2014-04-01

**Authors:** S Tomanović, S Ćakić, Ž Radulović, D Mihaljica, R Sukara, M Milutinović, E Ružić-Sabljić

**Affiliations:** 1 Laboratory for Medical Entomology, Department for Parasitology, Center of Excellence for Toxoplasmosis and Medical Entomology, Institute for Medical Research, University of Belgrade, Belgrade; 2 Institute of Microbiology and Immunology, Medical Faculty, University of Ljubljana, Ljubljana, Slovenia

## 

The geographic distribution of *Borrelia burgdorferi *sensu lato species in Europe shows very dynamic spatial and temporal variations. Since different *Borrelia *species usually correlate with different clinical manifestation of Lyme borreliosis, knowledge of the geographic distribution of the pathogen is very important for understanding the ecology and epidemiology of the disease. Our previous studies, based on direct molecular methods, revealed high diversity of species of the *B. burgdorferi *s.l. complex and unexpected high prevalence of *B. lusitaniae *in *Ixodes ricinus *ticks from Serbia. The aim of the present study was further isolation and typization of viable *B. burgdorferi *s.l. strains from vectors in Serbia as a basis for studies of biological, genetic and ecological variations.

A total of 248 adult *I. ricinus *ticks collected from 24 localities were processed for cultivation. Prior to cultivation, all ticks were disinfected, then triturated in BSK H medium. The culture tubes were incubated at 33°C for 3 months. Cultures were periodically examined by dark field microscopy. For confirmation of isolated strains, "seminested" PCR for the *fla*B gene was performed. Determination of *B. burgdorferi *s.l. species was carried out by the RFLP technique, using restriction enzymes *Mse*I and *Dra*I on the previously amplified 5-23S rDNA intergenic spacer (Postic et al., 1994). For *Borrelia *identification by RT-PCR targeting, the *hbb *gene protocol of Portnoï et al. (2006) was followed. Sequencing of the 5-23S rDNA intergenic spacer and *flaB *gene was performed for phylogenetic analysis.

Thirty-four spirochete cultures were isolated and subjected to further genotyping analyses. According to the RFLP patterns of the 5S-23S rDNA intergenic spacer, specimens were determined as: *B. lusitaniae, B. afzelii, B. burgdorferi *s.s., *B. garinii *and *B. valaisiana*. For all strains identified as *B. lusitaniae, B. garinii, B. afzelii *and *B. valaisiana *according to the *Mse*I-RFLP method, results of RT-PCR were in absolute agreement. However, Tm values for all strains identified as *B. burgdorferi *s.s. according to RFLP patterns were in the range for *B. lusitaniae *strains (64.17 to 64.58 °C). Thus, it was impossible to distinguish *B. burgdorferi *s.s. and *B. lusitaniae *strains by this method. Sequencing analysis for strains identified as *B. lusitaniae, B. garinii, B. afzelii *and *B. valaisiana *was in agreement with *Mse*I-RFLP and RT-PCR results. In cases of uncoordinated *Mse*I-RFLP and RT-PCR results, sequencing analysis confirmed unclear strains as *B. lusitaniae*. The results of this study showed *B. lusitaniae *to be the species with highest prevalence in *I. ricinus *ticks from Serbia.

